# Miscellaneous

**Published:** 1897-05

**Authors:** 


### MISCELLANEOUS.

### TREATMENT OF TRACHOMA.

All patients should use separate beds, towels, wash basins, etc. The general health should be looked after and all errors of refraction corrected. There is no known treatment that will rapidly cure firmly established cases of trachoma. When spasm of the orbicularis is present, canthotomy will give valuable aid. In acute manifestations, apply cold compresses frequently to the lids and irrigate the conjunctival sac with solutions of boric acid or sublimate (1 to 4,000 to 10,000).

As soon as the secretion is established use simple vaseline or ung. hyd. ox. rub. grs. ii., vaseline ʒii., to edge of lids at night. The strength (of the silver solution) usually adopted by the profession, varies from 1 to 5 per cent., which should be applied to the everted lids by means of absorbent cotton, from twice a week to twice a day, washing it off with a one per cent. salt solution. Many prefer the sublimate solution (1 in 100 to 500 water) used the same way.

When we have a full grown case of trachoma (chronic), the two remedies which have overshadowed all others from time immemorial, and which still have first rank, are nitrate of silver and sulphate of copper. Silver solution in the earlier stages when the secretion is more or less abundant, the cornea ulcerated and the conjunctiva succulent. When there is little inflammation and secretion and the cornea is intact, the case is better treated with a pencil of sulphate of copper thoroughly rubbed on all affected parts, from one to seven times a week. Many prefer the lapis divinus stick. Boro-glycerine, 30 to 50 per cent., is good in later stages. In mild cases tannin grs. 20 to 60 in one ounce of glycerine or alum stick to the lids are in favor.

In corneal complications use atropin and hot water, but when we have cured palpebral disease we are rid of the corneal.

Methods looking to the rapid destruction of the trachomatous bodies are expressions of the nodules by forceps of Knapp or Noyes. If the infiltration is deep-seated, the conjunctiva is scarified before expression.

"Grattage" or "Brossage" which consists in brushing briskly with a short stiff bristled tooth-brush the trachomatous conjunctiva, and afterwards applying a strong sublimate solution.

Curetting, excision of the individual nodules or of large portions of the retrotarsal fold; galvano-cautery to the separate neoplasms; electrolysis; scratching with special rakes, have had their advocates, —of these expression seems to be in greater favor by a majority. It is more generally applicable in follicular trachoma.—J. W. BULLARD, *Annals Ophthalmol. and Otology.*

---

### THE "FUROR SCRIBENDI."

Never, for a corresponding period in the history of medicine, has there been such a flood of technical literature as the last decade has produced. It is true that this decade has perhaps furnished more material than any that preceded it, but, even discounting this, there has been a veritable plethora of medical articles, monographs, and books. Foremost, perhaps, among the books we see a revival of the "systems"; a system of practice of medicine, of nervous diseases, of surgery, of gynecology, and so on. Doubtless this method is in a general way satisfactory, though it has many and serious defects.

Then the immense multitude of journal articles! a multitude no man, except the editor of the *Index Medicus*, can number. Nearly every city in this country boasts one or more medical journals, and the death of one seems to be the signal for two or three new ones to rise Phenix-like from its ashes. It is not clear whether the *furor*

*scribendi* is a cause or an effect of the multiplicity of medical journals. While most of the important work appears first in the journals, still there is such an immense amount of trash that the really valuable papers are often obscured.

A duty which is much neglected in our medical colleges is the training of the student in scientific writing. When he gets into practice and is attacked by the *furor scribendi*, he is very prone to choose a large subject. He is prone to revel in "typhoid fever," the "treatment of pulmonary tuberculosis, or "Bright's disease." If, instead of doing this, he would confine his ambition to some limited field—for example, instead of disposing of the whole subject of typhoid fever, he would take part of the subject, and discuss from the standpoint of his experience the eruption or the relapse, or some special symptom, his paper might be of value. Again, many valuable observations are obscured by the title. A "case of appendicitis" is usually passed over as one of no special interest, while in fact it may contain a point of great importance which is lost to the reader because it was not noted in the title. One who reads many journals gets into the habit, perhaps unconsciously, of passing over the papers on a general subject, unless he has reason to expect something from the particular author, and looks for special studies upon particular points. This concentration of work upon a limited area is what has made the monograph so valuable. If, then, the *furor scribendi* is to continue—and there is no indication of its subsidence—let it be directed to careful and accurate observation in a limited field. There are many men throughout the country who can, from time to time, furnish valuable microscopic bits, but it requires a master to make a subject interesting, macroscopically.—*Medical News.*

---

"A NEW METHOD OF DEALING WITH THE EXTERNAL MEATUS IN OPERATIONS UPON THE MASTOID."—(Lake, *Archives of Otology.*)

The author first mentions the various forms of procedure at present in use.

In the evolution of the mastoid operation an important advance was made by Stacke, who conceived the idea of throwing the attic, the antrum, and the tympanum into one cavity; at the same time he removed the ossicles and any remnants of diseased membrana tympani present. The next important step was that suggested by Zaufal, who removed the entire superior posterior wall of the bony meatus, and subsequently divided the external cartilaginous meatus along its posterior medial aspect. After Zaufal's came the operation as at present performed, and called the Panse-Körner operation. In this operation the external meatus (cartilaginous) was made to line the posterior and the external part of the cavity, and was either held in place by plugging through the enlarged external meatus or by stitches through it and the edge of the mastoid wound. The author performs his modification as follows: A longitudinal incision (No. 1) is made along the superior mesial part of the canal; another (No. 2) along the inferior mesial line parallel to the first. These two incisions pass through the cartilage of the meatus only, and are joined at their outer extremities by another (No 3) incision at the junction of the concha and meatus. The included cartilage is now removed. Incisions are now made through the skin of the meatus corresponding to those already made through the cartilages Nos. 1 and 3, and a flap is thus formed. The meatus is next replaced, and the post-auricular incision sewn up or not, according to the custom of the operator. The flap thus formed will be found to accommodate itself to the form of the cavity, which it will do, the resilient cartilage having been removed. The cavity thus formed is lightly plugged.—*Medical Chronicle.*

---

### FOREIGN BODIES IN THE EAR.

The following remarks by Dr. C. H. Burnett are intended for the guidance of the general practitioner: In regard to foreign bodies in the ear, the first and important injunction is to be sure that there is a foreign body in the ear before endeavors are made to extract it. Much damage has been done to the ear by groping after a foreign substance said to be in the ear which was not there and never had been. In no case should any one not a specialist use any form of surgical instrument to extract a foreign body from the ear. If a living insect has entered the ear, a few drops of sweet oil will smother it, and it may then be syringed out with warm water. If an inanimate substance has been placed in the ear, as is often done in the play of children, syringing with warm water will generally remove it if the ear has not been previously scratched by probes or forceps. If the latter has been done, the child should be etherized and the foreign body removed by an expert. There is no hurry demanded in such cases. The foreign substance had better be left in the ear indefinitely than to apply rough measures for its removal. What a child can slip into the ear in play can be easily removed if the physician first called knows how to do it. Unless he knows what to do and what not to do, he had better do nothing. It will be better to send the patient to the nearest aurist than to do the wrong thing for relief. Death has occurred by unskilful endeavors to remove a foreign body from the ear of a child. Not the foreign body in the ear, but the improper treatment, is the cause of death in such cases. The instillation of oil into the ear for the removal of foreign matter from it is futile. If, as sometimes happens in tropical countries, the larvæ of flies are present in the ear, a drop or two of chloroform or ether should be put into it.—*Practitioner.*

---

### RESULTS OF GYNECOLOGICAL AND ABDOMINAL SURGERY.

In answer to the question, "How are we to avoid peritoneal adhesions after laparotomy?" Dr. Byron Robinson, in *The Denver Medical Times* for October, remarks as follows:

"1. We must be as clean as possible, especially with hands and instruments.

"2. No irritating solutions, as bichloride, should be allowed to come in contact with the peritoneum.

"3. As little traumatism, and especially manipulation of the viscera, as possible should be practiced.

"4. We should use only absorbable ligatures. If infection occur in an absorbable ligature, it will only last a short time, for the absorption or breaking down of a ligature will end its action.

"5. No mucosa should be left exposed; the tubal ends should be covered with adjacent peritoneum or a graft of omentum. I never knew, in scores of experiments, a graft to die. All denuded parts of peritoneum should be covered with peritoneum. In the past two years Dr. Lucy Waite and I have assumed to remove the uterus in all cases of bilateral diseases, unless some contraindication existed, so that this avoids the presence of mucosa.

"6. Avoid using a drain-tube or gauze-packing if possible.

"7. Avoid peritoneal irrigation, as it spreads germs, desquamates the endothelium, and lays the grounds for peritoneal adhesions."—*American Medical Review.*

---

### HOW CHLOROFORM KILLS.

Despite the conclusions to the contrary of the two Hyderabad commissions, the general impression among medical men has always been that the dangerous or fatal effects of chloroform anesthesia are due to cardiac failure. As usual in such discussions, both schools have been partly right and partly in the wrong. Professor Hare, who has taken a great interest in this subject for many years, has proved conclusively (*Therap. Gaz.*, Feb. 15, 1897), we think, from original clinical and laboratory researches by himself and others, that the real primary cause of death in most cases is vaso-motor depression, "whereby the arterioles allow the blood to pass too freely into the great blood-vessel areas which are found in the capillaries and veins, and as a result the man is suddenly bled into his own vessels as effectually as if into a bowl." In fatal instances respiration usually ceases before the heart stops beating, because, on account of difference in position, the medulla becomes anemic before the heart. Here also lies the reason for the imminent perils that threaten when chloroform is administered in any but the recumbent position; it also explains the good effect of inversion and of abdominal compression when alarming symptoms supervene. The writer concludes, therefore, that in chloroform anesthesia the vaso-motor stimulant atropine ought to be given with the hypodermic syringe previously, and the head should always be kept low. When the case is feeble or already bloodless, bandage the limbs, and apply compresses deeply on the belly if the circulation begins to fail.—*Denver Med. Times.*

### FISTULA IN ANO.

Twelve cautions that should be observed in operating for fistula in ano:

1. Always operate under rigid aseptic conditions.

2. Be certain that all sinuses and diverticula have been divided.

3. See that the director is not forced out of the main tract into the neighboring tissues.

4. Divide the sphincter at a right angle, and not obliquely.

5. Ligature or twist all spurting vessels.

6. Guard against injuring the peritoneum when the sinus is high up.

7. Guard against cutting the vagina, prostate or urethra when the sinus is in the anterior wall of the rectum.

8. Do not operate on patients suffering from acute phthisis or Bright's disease.

9. Give patients the benefit of the sun as much as possible.

10. Do not pack the dressings tightly after the first twenty-four hours, but lay the gauze in the bottom of the tract.

11. Warn your patient of the possibility of incontinence following the operation.

12. Be guarded in your prognosis.—DR. S. G. GANT, in *Langsdale's Lancet.*

---

### TEN-CENT JOURNALISM.

It is not so very remarkable that certain medical publishers should be able to secure a list of subscribers when subscriptions are accepted for six months "on trial," for ten cents! But what must an advertiser think of the employment of such measures to pad a subscription list? Is it not confessedly a weakness on the part of the publisher to admit that he cannot get subscribers unless he accepts them at the paltry price of twenty cents a year? Surely he must have sample copies "to burn." Shades of the immortal Flint! Has it come to this, that the advertiser must not only pay for the space he occupies, but for the doctors' subscriptions as well? A strange part of it is that so many of the names of these "ten-cent" lists should have no initials prefixed. Not quite sure, evidently, of the correct names of all the "*bona fide*" ten-cent subscribers. Wonder what the Postoffice Department would say about a subscription list gained in this manner? The section of the postal laws pertaining to the admission of second-class matter in the mails distinctly states that the privilege shall be at once denied whenever it shall be shown that the publication is issued "primarily for advertising purposes, or if the subscription price is nominal." Would you call twenty cents "nominal"?—*Am. Med. Journalist.*

---

### SOME SUPPLEMENTARY REMARKS ON SCOPOLAMINE AS A MYDRIATIC.

Hydrobromate of scopolamine produces a complete paralysis of accommodation in sixty to ninety minutes, which persists at most only until the third day, and oftener only two. In simple refractive cases it is much to be preferred to atropine, which does not cause complete paralysis of accommodation until the second or third day and persists for from a week to ten days.

During the past year I have continued the use of this new mydriatic, and during this time no toxic symptoms have been manifested under its use except, perhaps, in half a dozen cases, and in these there was merely a slight indication of constitutional effects. I use it now, most frequently in a $\frac{1}{20}$ per cent. solution, and often only a $\frac{1}{40}$ per cent. solution. In special cases I sometimes employ it as strong as $\frac{1}{10}$ per cent. I use the weaker solutions with as few restrictions as I would use boracic acid, barring an unnecessary dilatation of the pupil. It produces no increase of tension and hence the old eye, even if it be glaucomatous, does not restrict its use, as in greater or less degree it would bar all other mydriatics.—DR. A. G. HOBBS, in *Ophthalmic Record.*

---

### TO FIND THE GONOCOCCUS.

Wyeth, in the *Polyclinic,* gives these directions: Place a small drop of the discharge upon a cover-glass and smear by rubbing two cover-glasses together; dry it by passing one of the cover-glasses with pus side upward through a spirit-flame two or three times; immerse this at once in a solution of methyl-blue; wash off the excess of coloring matter by holding it under clear running water or by dipping the glass several times into clear water; dry the stained pus well by pressing with blotting paper; then cover it with a small drop of cedar oil; put on a thin cover-glass and examine with a lens magnifying from 700 to 1,000 diameters. The peculiar double-bean shaped arrangement of the diplococci will be seen within the protoplasm of the pus corpuscle and epithelium.

---

### THE PREVENTION OF IODISM IN THE USE OF POTASSIUM IODIDE.

Spencer (*Journal de médecine de Paris*, March 28, 1897) is credited with the following formula:

| | | |
|---|---|---|
| ℞ | Potassium iodide | 30 parts. |
| | Ammonium ferrocitrate | 4 parts. |
| | Tincture of nux vomica | 8 parts. |
| | Distilled water | 30 parts. |
| | Tincture of cinchona, enough to make | 120 parts. |

M. S.: A teaspoonful in half a glass of water, to be taken after each meal. The tincture of nux vomica and the ammoniocitrate of iron are said to check the tendency to coryza and at the same time to act as tonics.—*New York Medical Journal.*

---

WHEN a wound, either accidental or operative, shows signs of infection, never wait for suppuration. Immediate incision, thorough disinfection, and drainage, if necessary, relieve pain, shorten the duration and prevent extension of the inflammatory process. In draining a suppurating wound, never cork it up by packing gauze in it. The smallest strip that will reach the bottom of the cavity, very loosely applied, is the best. Constitutional treatment is all-important in all forms of diffuse surgical inflammation. Recurrence of carbuncles and boils suggests an examination of the urine for diabetes. See that patients have a good night's sleep before an operation. Skin-grafting will not succeed upon an unhealthy surface. Watch patients with burns of the pharynx and larynx; be ready to operate at once. Severe dyspnea may occur with appalling suddenness. If the patient is getting cold and feeble, his ability to feel pain has greatly diminished. Waste no time in anesthesia in emergency tracheotomies.—*Ex.*

